# Sublethal Exposure to Diatomaceous Earth Increases Net Fecundity of Flour Beetles (*Tribolium confusum*) by Inhibiting Egg Cannibalism

**DOI:** 10.1371/journal.pone.0088500

**Published:** 2014-02-07

**Authors:** Allen W. Shostak

**Affiliations:** Department of Biological Sciences, University of Alberta, Edmonton, Alberta, Canada; USDA-Agricultural Research Service, United States of America

## Abstract

Population regulation results from an interplay of numerous intrinsic and external factors, and for many insects cannibalism is such a factor. This study confirms a previously-reported observation that sublethal exposure to the fossilized remains of diatoms (i.e. diatomaceous earth) increases net fecundity (eggs produced minus eggs destroyed/day) of flour beetles, *Tribolium confusum*. The aim was to experimentally test two non-mutually-exclusive ecological mechanisms potentially responsible for the increased net fecundity: higher egg production and lower egg cannibalism. Adult *T. confusum* were maintained at low or high density in medium containing sublethal (0–4%) diatomaceous earth. Net fecundity increased up to 2.1× control values during diatomaceous earth exposure, and returned to control levels following removal from diatomaceous earth. Cannibalism assays on adults showed that diatomaceous earth reduced the number of eggs produced to 0.7× control values at low density and to 0.8× controls at high density, and also reduced egg cannibalism rates of adults to as little as 0.4× control values, but at high density only. Diatomaceous earth also reduced cannibalism by larvae on eggs to 0.3× control values. So, while the presence of diatomaceous earth reduced egg production, net fecundity increased as a result of strong suppression of the normal egg cannibalism by adults and larvae that occurs at high beetle density. Undisturbed cultures containing sublethal diatomaceous earth concentrations reached higher population densities than diatomaceous earth-free controls. Cohort studies on survival from egg to adult indicated that this population increase was due largely to decreased egg cannibalism by adult females. This is the first report of inhibition of egg cannibalism by diatomaceous earth on larval or adult insects. The ability of diatomaceous earth to alter cannibalism behavior without causing mortality makes it an ideal investigative tool for cannibalism studies.

## Introduction

Cannibalism, or intraspecific predation, occurs in various invertebrate and vertebrate taxa [1], [2] and is considered to be a normal response to a variety of environmental situations. The intensity of cannibalism may vary under stressful conditions, and it can reduce population size before acute resource limitations occur [2]. Cannibalism is widespread among insects in general and is particularly common in beetles [3]. Beetles (Insecta: Coleoptera) of the genus *Tribolium* have been used as model systems for ecological and evolutionary study since the work of Chapman [4], and cannibalism by adults and larvae on all life stages plays a role in controlling population growth [5]–[7]. Egg cannibalism appears to be a particularly potent mechanism, and it may remove up to 98% of eggs that are laid [6]. Among adult beetles, females have a much higher rate of cannibalism on eggs than do males [6]. Among larvae, egg cannibalism rates increase as larvae age, declining only just prior to pupation [8]. Egg cannibalism in beetles does not necessarily fine-tune the population to available resource levels, and in fact may reduce the population well below environmental carrying capacity [9]. The effect of cannibalism on egg numbers has made it important to distinguish between “real fecundity”, which is the rate at which eggs are actually oviposited under a given set of environmental conditions, and “net fecundity”, which is eggs produced minus eggs destroyed/day [5]. The ecological mechanisms for regulating population density may, in turn, be modified by intrinsic factors such as beetle genetics [10], sex [11], [12], and age [6], and by extrinsic factors such as beetle density [6], [11], environmental conditions [13], [14], and the presence of parasitism [15].

One environmental factor potentially impacting population growth is the presence of stressors such as diatomaceous earth (DE), which is mined from the fossilized remains of diatoms and is widely used as a “natural” insecticide [16]. Consisting almost entirely of SiO_2_, DE is believed to function by abrading or adsorbing lipids from the epicuticle of insects, leading to desiccation and death [17]. DE is considered relatively safe from a human health standpoint [17], and its potential as an insecticide has been studied on a variety of insect pests [17]–[20]. Studies on insects following DE application to whole grain kernels typically report reduced production of progeny [21]–[23]. By contrast, a recent study [24], which examined whether DE in flour might act as an environmental stressor on flour beetles (*Tribolium confusum*), made an incidental observation that young adult beetles exposed to a sublethal concentration of DE accumulated up to 6× more beetle eggs in the medium compared to beetles maintained in the absence of DE. This apparent increase in net fecundity occurred only while DE was present and did not persist after removal of the insect from DE [24]. The possibility that an insecticide might not just fail to kill the target insect at low concentrations, but actually increase its population size by increasing net fecundity, could have serious practical implications for pest control. Notwithstanding the practical issues, this observation also raises basic biological questions on the underlying mechanism(s): did the observed increase in net fecundity in the presence of sublethal DE result from an increase in real fecundity, a decrease in egg mortality from cannibalism by adult beetles, or some combination of the two? Moreover, was this unusual observation restricted to the limited range of experimental conditions used previously [24], or was it indicative of a more broadly expressed response of these beetles to DE as an environmental stressor? Studies on sublethal pesticide effects on other arthropods typically report reduced fecundity [25]–[29], but increased fecundity sometimes occurs [30] and the same pesticide can cause opposite responses in different organisms [25], [30]. I have found no reports linking sublethal pesticide exposure to decreased cannibalism.

Beetle age is an important factor in the fecundity of *T. confusum*, yet the effect of DE exposure on beetles of different ages has received little attention. Real fecundity and cannibalism by adults on eggs both increase rapidly during the first 1–2 weeks post-eclosion, and decline at all densities as beetles age beyond 30–60 days post-eclosion, but rates/individual beetle for both measures are progressively lower as densities increase [6], [31]. At the population level, cultures of *T. confusum* initiated with a small number of adults, particularly young adults, typically overshoot their equilibrium population size during the first 60 days of culture, when the adult population still consists entirely of F_1_ individuals [31], [32]. The mortality of *Tribolium castaneum* following DE exposure is sharply higher in 64-day-old beetles than in 4–32-day-old beetles [33]. These observations suggest that any sublethal effects of DE on fecundity of *T. confusum* would be most evident in younger beetles.

This study had the following aims: (i) to experimentally test the effects of various DE concentrations on net fecundity under a range of adult beetle ages and densities; (ii) to test two non-mutually exclusive hypotheses on the ecological mechanism responsible for increased net fecundity, i.e. whether DE acts on adult *T. confusum* by increasing egg production and/or by reducing egg cannibalism; (iii) to assess the consequences of sublethal DE exposure for early population growth of *T. confusum*. All these aims were achieved.

## Materials and Methods

### Beetles

No specific permits were required for the collection and maintenance of insects and the subsequent laboratory studies. *Tribolium confusum* were routinely maintained in mass cultures in flour medium (unbleached white flour supplemented with 5% weight/weight (w/w) brewer’s yeast) and stored in the dark in an incubator at 27–28°C and 9–20% ambient relative humidity. Although lower than the humidity used by other researchers, this is the ambient humidity in which these beetle cultures have been maintained for >15 years. Densities of adult beetles were about 3–6/g medium each time fresh cultures were established and were about 6–15/g medium when cultures were changed (every 2–3 months). These are densities that still allow the population to be in a growth phase [4] although likely influenced by both egg cannibalism and reduced real fecundity [6]. Pupae collected from the mass cultures for use in experiments were sexed and held separately until eclosion, and then the adults were stored in the incubator, in known-age, same-sex groups at a density of 10–15/g medium until use. Sample sizes were selected to maximize usage of all purpose-reared beetles.

Under these conditions, newly-laid eggs hatch in about 6 days. The first pupae are observed as early as 4–5 weeks following initiation of a new culture, but appearance of large numbers of pupae usually takes 5–6 weeks. The pupal stage typically lasts 1–2 weeks. Following eclosion, adults take several days to 2 weeks to commence oviposition. This colony of *T. confusum* normally has low mortality until about 30 weeks post-eclosion, and thereafter exhibits rapidly increasing mortality and declining net fecundity, although some individuals live >1 year [34]. These observations are within the range of expectations for this species [35].

### Test Media, Apparatus and Design

Experiments were conducted in flour medium alone (control), or flour medium supplemented with various concentrations of diatomaceous earth (DE) (Insect Stop®, Aerokure International Inc.; 91.1% SiO_2_). In all cases, mixing of DE in the medium was done by shaking for at least 2 minutes, passage through a 250 µm mesh sieve, and shaking again for an additional 2 minutes. Experiments were conducted using 3 g (for high beetle density treatments) or 12 g (for low beetle density treatments or for multi-week experiments) of test media in either 15 ml Wheaton vials (25×30 mm) or 18×150 mm test tubes with a foam stopper. The same type of container was used within each experiment.

Experiments were typically initiated with 5 virgin female and 5 virgin male beetles of similar age (maximum age range = 1 week)/container, placed together for the first time at the start of each experiment. Where experiments used multiple beetles/container, each group was assembled haphazardly from its source stock, but treatments were assigned to the containers at random. Within the broad goals of the experimental program, the choice of specific beetle ages for testing in each experiment was based on availability of sufficient numbers of beetles at the time an experiment was done. Each experiment used age-matched controls. All experimental set up and monitoring was done under lighted, bench-top conditions, within the period 07∶00–17∶00 h, with animals removed from, and returned to, incubator conditions as a group.

### Experiment 1: Time Course of DE Action on Adults

Two experiments were conducted to examine the time course of DE action on adult survival and net fecundity (*Nt)*. Specifically, the aim was to assess the dose-response of adult beetles to DE and to determine the range of DE that might be considered sublethal.

#### Experiment 1a

Five female and 5 male newly-emerged beetles were placed/vial containing 3 g medium and 0%, 1%, 2%, 4%, 8% or 16% DE (w/w). There were n = 4 replicates/treatment. At 5-day intervals until day 30, a census was made of numbers of adult beetles (live and dead) and eggs in the medium, and the medium was replaced with fresh medium of the same DE concentration. Dead adult beetles were removed for sexing (placed in 10% KOH at 60°C for 2–3 hours to clear and soften, then mounted under a cover glass to observe genitalia). The medium in all vials was replaced with DE-free medium on day 30 to assess recovery of all groups from DE exposure. At 5-day intervals until day 40, a census of egg and adult beetle numbers was performed, and the medium was replaced with fresh DE-free medium. The replacement of medium every 5 days helped to maintain initial DE concentrations and to remove eggs prior to hatching so that egg counts would not be confounded by egg cannibalism by newly-emerged larvae.

#### Experiment 1b

This experiment was similar to experiment 1a, with the addition of host age as a second treatment factor. Three ages of adult beetles were tested (0, 12 and 24 weeks post-eclosion), which spans the normal period of high adult survival and high fecundity [31]. DE concentrations were limited to 0%, 1% and 4% because experiment 1a showed that ≥8% DE soon caused significant mortality. There were n = 4 replicates/treatment combination. Censuses in the presence of DE were done at 5 day intervals until day 25. Then, beetles were transferred to DE-free medium to assess recovery from DE exposure using 5-day census intervals from days 25–40.

### Experiment 2: Effect of DE on Egg Cannibalism by Adults

Egg cannibalism by adults was tested using a modified marked-egg method [6], [11]. This method measures cannibalism by the rate of disappearance of previously-added marked eggs and appearance of newly-laid unmarked eggs, under the assumption that the beetles cannibalize both marked and unmarked eggs at a similar rate. Mixed adult beetles from the mass cultures were added to containers with 200 g medium (0.5–1 beetle/g) dyed with neutral red crystals, 0.3% (w/w). Because *Tribolium* spp. eggs are laid with a sticky surface to which flour readily adheres, most eggs became coated with dyed medium; uncoated eggs were discarded. On the day of an assay, groups of 100 dyed eggs were added to normal, non-dyed medium. The medium was shaken side-to-side to disperse the eggs, and then poured through a funnel into a test tube. Adult beetles were then added to the medium; any newly-laid eggs would become coated only with non-dyed medium. A preliminary trial was conducted to verify the recovery of dyed eggs in the absence of adults. Ten samples of 100 dyed eggs were prepared and randomly allocated to either 12 g medium (n = 5) or 12 g medium containing 4% DE (n = 5) in the same manner as in the cannibalism assays. After 24 hours, the medium was sifted and eggs were recovered and counted. In the absence of adults, 100% of eggs were recovered in all 10 samples.

Another preliminary experiment (data not shown) indicated that up to 40% of dyed eggs in 3 g medium with 10 adults were eaten within 1 day. Therefore, 1 day was chosen as the length of the cannibalism assay because a longer assay would reduce the ability to discriminate among treatments. Egg cannibalism by *Tribolium* spp. varies with beetle and egg density, the duration of the observation period, and even prior cannibalism experience [6], [11]. The design of experiment 2 used beetle densities comparable to those in other experiments, so that real fecundity and cannibalism rates could be determined and readily interpreted in view of results from other parts of this study. A repeated measures design was used in which an initial group of adult beetles was tested in a “baseline round” of handling and observation to determine their egg production and cannibalism behavior at different beetle densities in the absence of DE. Then, during a subsequent “treatment round”, those same beetles were assigned to different concentrations of DE, but otherwise subject to the same sequence of handling and observation as in the baseline round.

One day prior to the start of the experiment, 5 virgin female and 5 virgin male 3–4-week-old beetles were added to 42 vials containing 3 g DE-free medium to permit mating. On day 0, the baseline round began and these beetles were removed from their vials and added to test tubes containing 3 g (high beetle density, n = 21 replicates) or 12 g (low beetle density, n = 21 replicates) DE-free medium. On day 5, a census was made of beetle and egg numbers. This produced an estimate of *Nt* averaged over the 5-day period from days 0–5, and the ability to compare to results of 5-day-long *Nt* assays from experiment 1. Then, beetles were transferred to clean test tubes containing the same amount of fresh medium for a 1-day transition period, to reduce any effects of the beetles’ previous cannibalism experience (days 0–5) on their performance during the subsequent 1-day cannibalism assay (days 6–7).

On day 6, the baseline cannibalism assay was performed. Beetles were transferred to clean test tubes containing the same quantity of fresh medium as before, plus 100 dyed eggs. On day 7, a census was made of beetle numbers and of the numbers of dyed and non-dyed eggs. Sonleitner’s [6] modification of a model by Rich [11] was used to produce an estimate for *Nt*, expressed herein as the total number of non-dyed eggs accumulating/female beetle/day; *E* (real fecundity), the number of eggs oviposited/female beetle/day; and *c* (cannibalism rate), the fraction of eggs eaten/beetle/day.

In the treatment round, which followed the cannibalism assay in the DE-free baseline round, the same 42 tubes of beetles from the baseline round were allocated at random to three DE treatments while using the same quantity of medium as in the baseline round (0%, 1% or 4% DE; n = 7 replicates/combination of DE and beetle density). The same sequence of procedures was applied as in the baseline round, but this time always using the assigned density and DE concentration: storage for 5 days in medium (days 7–12), census of adult beetle and egg numbers, transfer to a new vial for a 1-day transition period in medium lacking dyed eggs (days 12–13), and finally transfer to medium containing dyed eggs for the treatment round cannibalism assay (days 13–14).

### Experiment 3: Population Growth in the Presence of DE

This experiment evaluated the response of a newly-started and undisturbed beetle culture to the continuous presence of DE, and the effects on early population growth through to the emergence of adult F_1_. For each replicate, 5 female and 5 male newly-emerged beetles were placed/test tube containing 12 g flour medium with DE in the following concentrations: 0%, 0.5%, 1%, 2%, 4% and 8%. Sufficient samples were established to allow for a final census following undisturbed maintenance for 42, 56 or 70 days (n = 4 replicates/day and/DE concentration). Day 42 was anticipated to provide an observation as the first of the F_1_ adults were emerging, and day 70 was expected to provide an observation after the last of the F_1_ adults emerged, but before any F_2_ adults should be present. During each census, medium was passed through a 600 µm mesh sieve. Larvae that passed through following a consistent agitation period (20 sharp raps with a hand against the side of the sieve over 10 seconds) were classified as “small” larvae; those retained in the sieve were classified as “large” larvae. Small larvae were typically <3 mm long, and large larvae were typically >3 mm long, although the distinction was not absolute. Small larvae would be expected to be instar 1–3, and <1 week post-hatching [32]. Each culture was censused only once.

### Experiment 4: Egg Hatching Success in the Presence of DE

Egg hatching success was assessed in 2 experiments to provide data for interpretation of the population growth study (experiment 3).

#### Experiment 4a

This experiment produced eggs that became directly coated with the medium–DE mixture as they were laid, like experiment 3. Adult beetles (mixed sex) from the main colony were placed in the incubator for 18 hours at a density of 1 beetle/5 g medium containing 0%, 1%, 4% or 8% DE. Then, eggs from each source were collected, examined to eliminate any showing obvious signs of damage or deformity, placed in random order 1/vial containing 1 g of corresponding medium (n = 36 replicates/DE treatment), and stored in the incubator for 10 days to give all eggs ample opportunity to hatch. On day 10, the contents of each vial were passed through a 180 µm mesh sieve and the egg was examined for signs of hatching.

#### Experiment 4b

This experiment produced eggs that became directly coated with DE-free medium as they were laid, but were then stored in medium containing various concentrations of DE so that larvae on hatching would be in direct contact with DE. This enabled a single source of eggs to be collected and then randomly allocated to DE treatments, so that the larvae hatching from them could also be used in cohort survival studies (experiments 6a and 6b). Eggs were collected from adult beetles (mixed age and sex) following 6 hours storage at a density of 1 beetle/10 g medium with 0% DE. Individual eggs were allocated at random to vials containing 1 g medium with 0%, 1% or 4% DE, and stored under standard conditions in an incubator. In one trial (n = 24 replicates/DE treatment), done in conjunction with experiment 6a, each egg was examined daily for signs of hatching after passing vial contents through a 180 µm sieve and then placing it into fresh medium. In a second trial (n = 48 replicates/DE treatment), done in conjunction with experiment 6b, each egg was first examined 14 days post-laying to minimize pre-hatch handling.

### Experiment 5: Effect of DE on Egg Cannibalism by Larvae

The population growth study (experiment 3) was sufficiently long to allow eggs to hatch and develop through all larval stages. As with adults, larvae *T. confusum* are also egg cannibals [8]. This experiment was designed to test whether exposure to DE over time affected egg cannibalism by larvae.

Larvae were isolated from a stock culture of *T. confusum* and divided into two groups. One group was placed in medium with 0% DE, the other in 4% DE, and they were stored in the incubator under standard conditions for 5 days. After this pre-treatment, larvae were separated into length classes 3–4.9 mm, 5–5.9 mm, and 6–6.9 mm. These classes would include larval instars ≥4 [35] which would be expected to exhibit the greatest egg cannibalism [7]. Larvae were separated into groups of 10/test tube containing 3 g medium with the same DE concentration as their pre-treatment, plus 100 freshly-collected eggs. There were n = 4 replicates/DE concentration in length classes 3–4.9 mm and 5–5.9 mm, and n = 10 replicates/DE concentration in length class 6–6.9 mm. After 24 hours, the number of eggs remaining and larval survival were censused. These conditions approximated the high beetle density, high DE concentration, conditions used in the adult egg cannibalism assay (experiment 2).

### Experiment 6: Survival of a Cohort of Eggs under Various Egg Cannibalism Scenarios

Population growth in the presence of DE (experiment 3) might be affected by mechanisms other than cannibalism. It would be helpful to evaluate the sources of cannibalism vs. non-cannibalism mortality for a cohort of eggs laid under the conditions of experiment 3. However, the continual production of eggs by the adults in those cultures, and the larvae hatching from them, would render inaccurate and impractical any attempts to track the fate of an initial cohort of eggs. Therefore, a stepwise approach was adopted. First, the fate of eggs maintained individually, i.e. in the absence of cannibalism, was evaluated. Then, the fate of eggs maintained in groups was tracked, where the only source of cannibalism could be by larvae hatching from those eggs. With near-synchronous hatching of eggs expected, cannibalism by early-hatching larvae on late-hatching eggs was possible, but expected to be negligible, and the main opportunity for cannibalism would be larva-on-larva. Finally, the fate of eggs maintained in groups, but also in the presence of virgin male or female adult beetles, was tracked. In the latter scenario there would still be the potential for cannibalism by larvae hatching from the eggs, but in addition there could be ongoing cannibalism on eggs or larvae by the virgin adults, without the complications arising from continuous introduction of new viable eggs that would occur if mated adults were present.

#### Experiment 6a

This experiment was conducted to determine development and survival of a cohort of eggs maintained individually in the presence of DE. Using eggs from the first trial of experiment 4b (n = 24 eggs at each of 0%, 1%, and 4% DE), each vial was examined daily by passing the contents through a 180 µm sieve and characterizing the contents (hatched or unhatched egg, live or dead larva, pupa or adult). Any molted exoskeletons present were recorded to establish number and timing of molts. Lengths were recorded for live and dead larvae. Sex was determined at the pupa stage. Hatched eggs, molted exoskeletons, and dead beetles were discarded. Live beetles were placed in fresh medium. When the cuticle had hardened, 2–3 days after the final molt, adults were killed, and the length of the left elytrum was measured as an index of body size. The use of single eggs eliminated opportunities for cannibalism, and the final stage reached by any dead individuals could be determined.

#### Experiment 6b

This experiment replicated the basic design of experiment 6a, but used eggs from the second trial of experiment 4b (n = 48 eggs at each of 0%, 1%, and 4% DE), maintained individually, with vials censused only at 14-day intervals up to 42 days. This design was employed to reduce the minor handling loss (9/66) of small larvae that occurred during the daily examinations in experiment 6a (see results for details). Examination on day 14 provided observations when most beetles were “small larvae” (see experiment 3), while day 28 provided observations on “large larvae” (experiment 3) nearing pupation, and day 42 provided observations mainly on newly-emerged adults, and enabled a direct comparison with the day 42 census done in experiment 3.

#### Experiment 6c

This experiment was conducted to determine survival of a cohort of eggs maintained in groups in the absence of adults. The design was similar to experiment 6b, but used groups of 100 eggs/test tube containing 12 g medium with 0%, 1% or 4% DE. There were n = 4 replicates/treatment. A census was conducted on days 14, 28 and 42. After each census, dead beetles were removed and all live beetles, feces and molted exoskeletons recovered from the tube, were added into 12 g fresh medium to attempt to recreate conditions as they were prior to the census. However, the old medium was not re-used because full recovery was not practical, and some loss of DE during sieving was expected.

#### Experiment 6d

This experiment was conducted to determine survival of a cohort of eggs maintained in groups and in the presence of virgin adults. The basic design of experiment 6c was used, but with groups of 200 eggs/test tube containing 12 g medium with 0%, 1% or 4% DE. Groups of 10 virgin males or 10 virgin females, 2–3 weeks post-eclosion and pre-treated for 3 days in the corresponding DE concentration, were also added to each tube. There were n = 3 replicates/treatment combination of DE concentration and beetle sex. This design replicated the initial total mass of medium and low adult beetle density used in experiments 2 and 3, with an initial egg density that would approximate the ongoing number of unhatched eggs expected to be present in experiment 3 (5 females/tube, laying 8 eggs/female/day, and taking 5 days to hatch). A census was conducted at days 14, 28 and 42.

### Data Analysis

Statistical analyses were performed using SAS (version 9.2, SAS Institute Inc.). Beetle survival was tested using the life table method (PROC LIFETEST). Data were right-censored after 40 days (experiment 1) or 42 days (experiment 6). Multiple comparisons of survivorship functions used the log-rank test, and 95% CI on hazard rates were used to evaluate interval-specific mortality.

Beetle population size, egg production, and cannibalism rates were tested by analysis of variance (ANOVA). Each vial or tube of beetles was treated as a unit of independent replication. Assumptions of normality were evaluated by visual examination of residual plots and homogeneity of variances by Levene’s test. Data were transformed where necessary to meet these assumptions. Net egg production (experiment 1) and cannibalism of eggs by adults (experiment 2) were tested using a repeated-measures, mixed-model design (PROC MIXED). Selection of covariance structure was based on the methods of Littell et al. [36] and used a combination of graphical analysis and information criteria (Akaike Information Criterion, AIC; Schwarz’s Bayesian Criterion, SBC). In most cases, the choice of covariance structure made no difference to the statistical conclusions; where there was a difference between AIC and SBC, the simpler model was chosen. Population size (experiment 3), cannibalism of eggs by larvae (experiment 5), and development time and cannibalism in cohort studies (experiment 6) were analyzed by one-way or factorial ANOVA (PROC GLM).

Population stage-structure (experiment 3) was tested using ordered logistic regression (PROC LOGISTIC). Hatching success (experiment 4) was tested by contingency table analysis (PROC FREQ) or logistic regression (PROC LOGISTIC). Stage-specific development time categories (experiment 6a) were compared using the Mantel-Haenszel test (PROC FREQ).

Post-hoc, multiple pairwise comparisons used the Tukey adjustment. Selection of minimal display letter combinations for pairwise comparisons was based on a published algorithm [37]. Statistical significance was determined using α = 0.05.

## Results

### Time Course of DE Action on Adults

#### Experiment 1a

**Figure 1:**
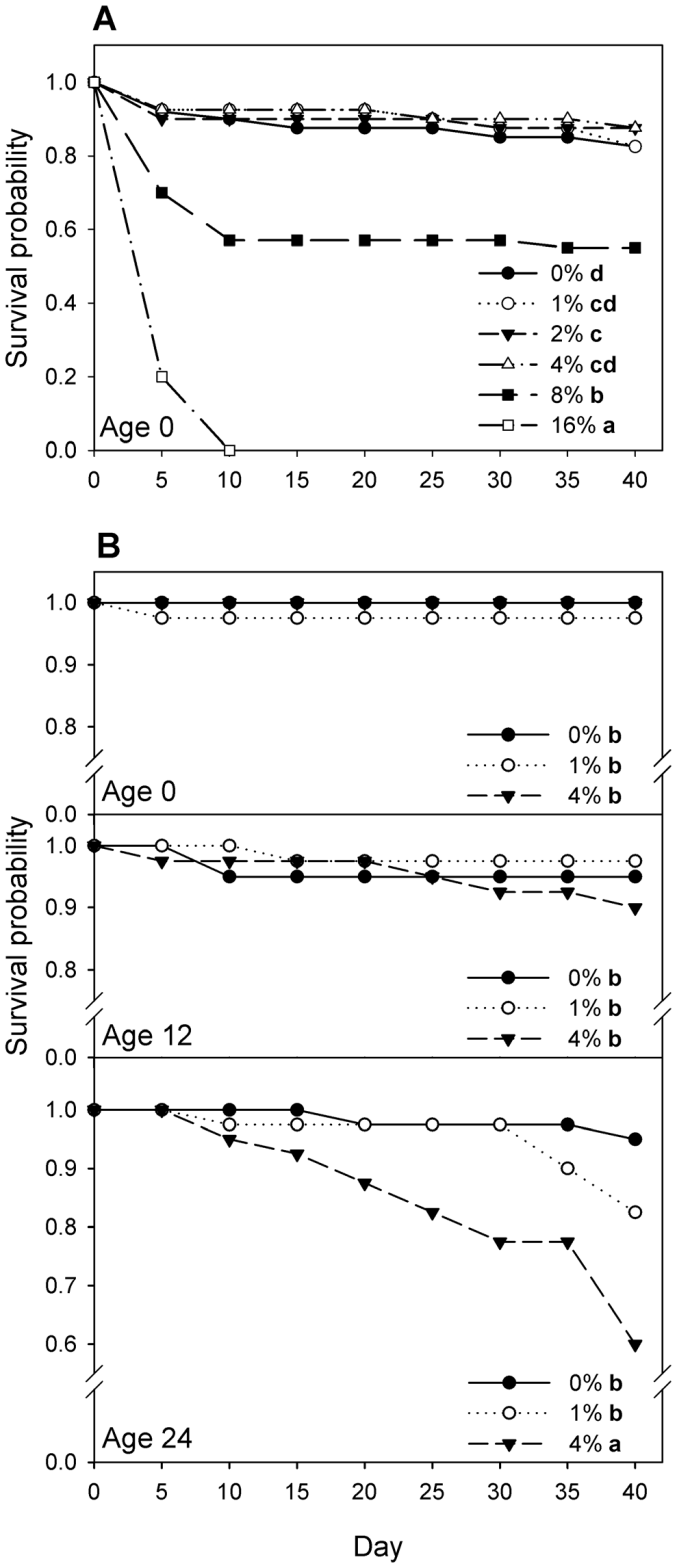
Survival of *Tribolium confusum* exposed to diatomaceous earth. Life table survival functions for adult beetles exposed continuously to different concentrations of diatomaceous earth (DE). (A) Beetles initially 0 weeks old (experiment 1a). (B) Beetles initially 0, 12 or 24 weeks old (experiment 1b). Within (A), or among all panels of (B), survival functions for labeled DE concentrations followed by the same letter do not differ significantly (Tukey multiple comparison adjustment).

Newly-emerged (0 week old) adult beetles exhibited a significant DE-concentration-dependent decrease (log-rank test of equality over strata, χ^2^_5_ = 144, p<0.001) in survival (Fig. 1A). Pairwise tests indicated that survival was statistically indistinguishable among concentrations 0–4% DE (Fig. 1A), where collectively only 24/160 (15%) died by day 40. Survival was significantly lower in 8% DE, with a further significant decrease in 16% DE (Fig. 1A).

**Figure 2:**
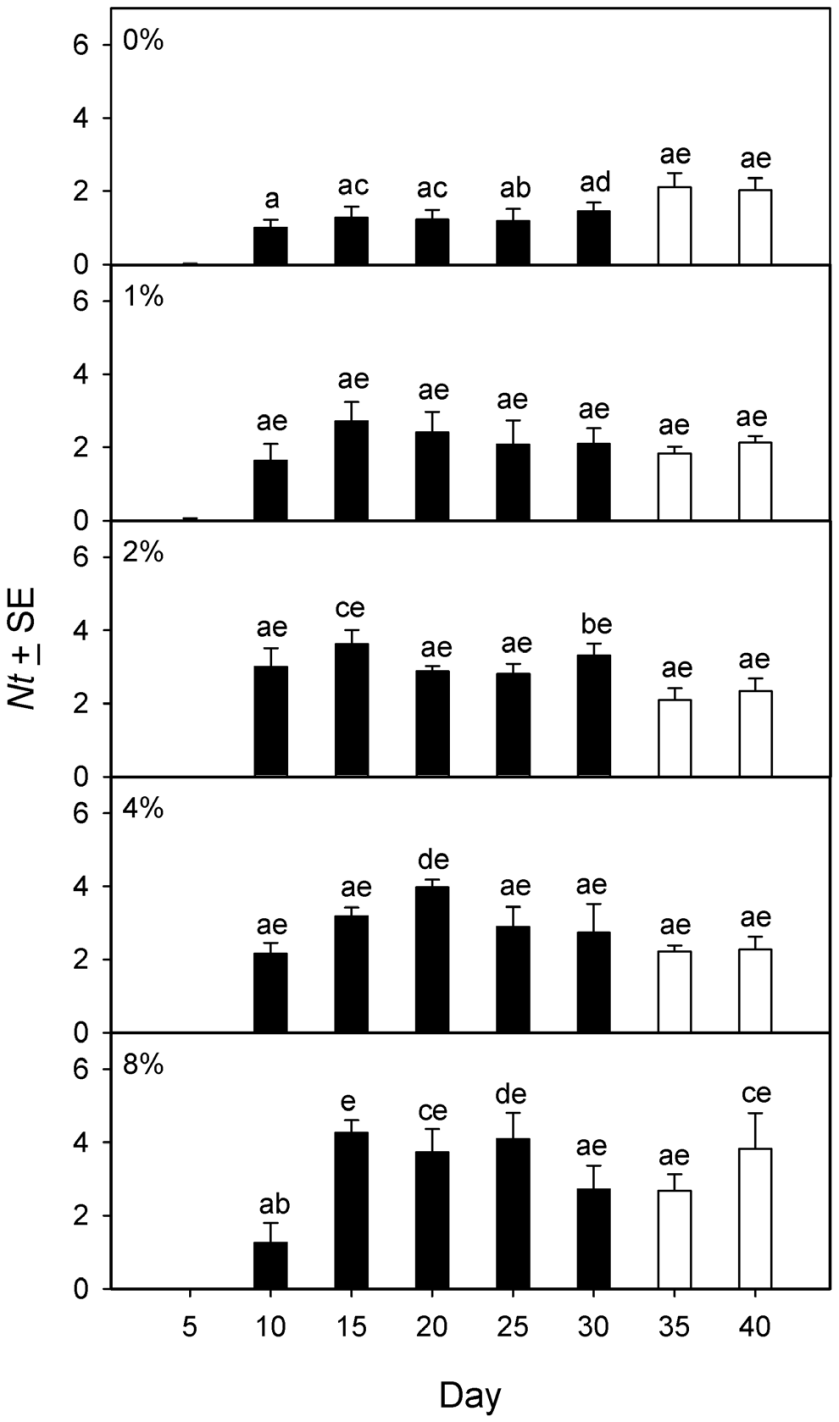
Net fecundity of *Tribolium confusum* exposed to diatomaceous earth. Net fecundity (*Nt*), expressed as the mean daily accumulation of eggs/adult female beetle over successive 5-day census intervals in different concentrations of diatomaceous earth (DE) (experiment 1a). After the day 30 census (white bars), all treatment groups were stored only in 0% DE. Across all panels, means with the same letter do not differ significantly (Tukey multiple comparison adjustment).

Newly-emerged adult beetles produced few eggs during the first census interval, but thereafter produced eggs in all censuses at all DE concentrations (Fig. 2). Net egg production *Nt* from each 5-day census interval was standardized/female/day, log-transformed, and analyzed using PROC MIXED with an autoregressive covariance structure, DE concentration as the fixed effect, and census interval as the repeated measure. There was a significant interaction between DE concentration and census time (F_4,15_ = 3.14, p = 0.046). Pairwise comparisons (Fig. 2) indicated that *Nt* under control conditions (0% DE) did not change over the course of the experiment, but that *Nt* at 2%, 4% or 8% DE was significantly higher than control for each census on days 15–30. Following the return of all beetles to DE-free medium after the day 30 census, *Nt* in all groups of beetles previously exposed to DE was indistinguishable from the DE-free control for the corresponding census period (Fig. 2).

#### Experiment 1b

Adult beetles exhibited significant (log-rank test of equality over strata, χ^2^_8_ = 67, p<0.001) variation in survivorship among age group×DE concentration combinations (Fig. 1B). Pairwise tests indicated that survival was statistically indistinguishable among 0-week-old and 12-week-old beetles at 0–4% DE and 24-week-old beetles at 0–1% DE, where collectively only 17/320 (5%) died by day 40 (Fig. 1B). Only the 24-week-old beetles in 4% DE had significantly lower survival than all other groups (Fig. 1B).

**Figure 3:**
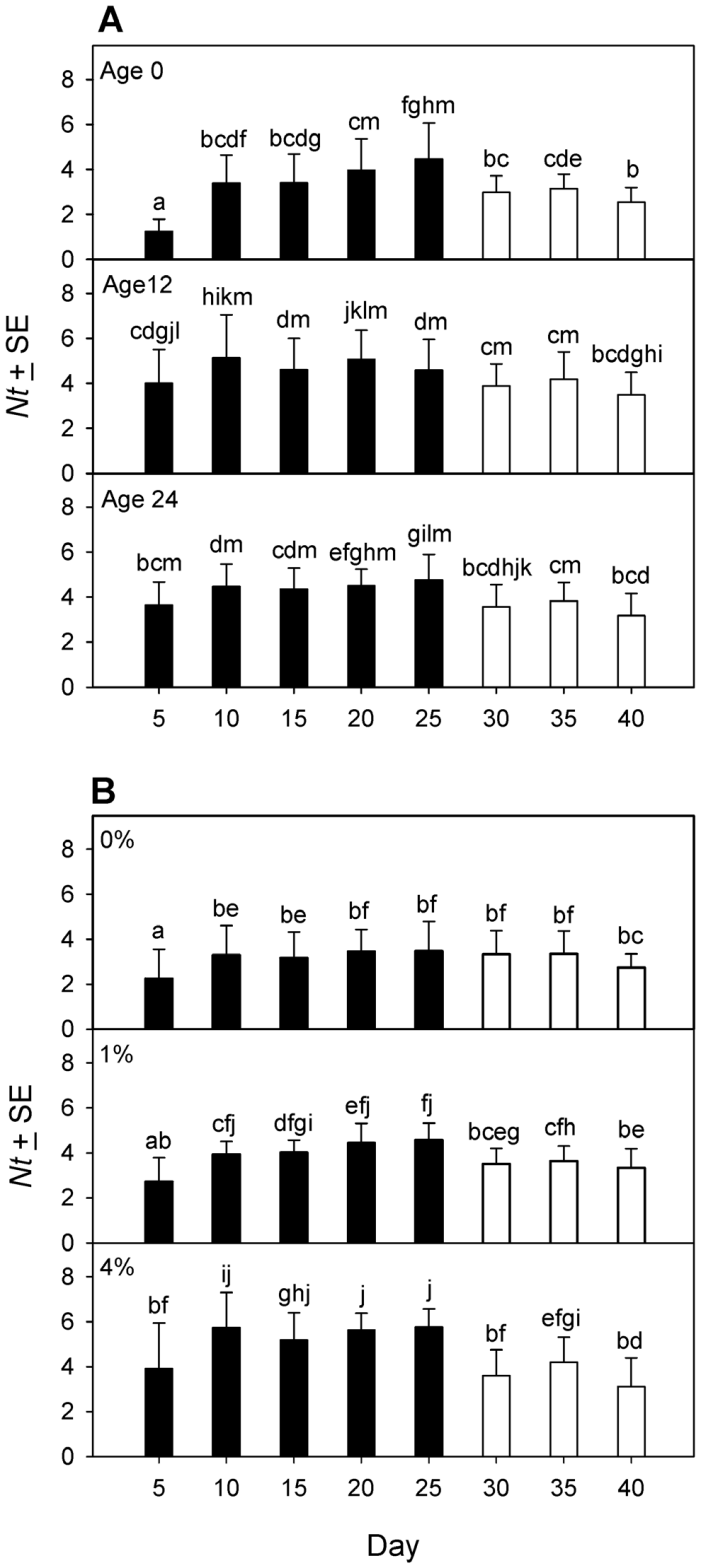
Effect of age and diatomaceous earth on net fecundity of *Tribolium confusum*. Net fecundity (*Nt*), expressed as the mean daily accumulation of eggs/adult female beetle over successive 5-day census intervals. (A) Different age classes of beetles (experiment 1b). (B) Different concentrations of diatomaceous earth (DE) (experiment 1b). After the day 25 census (white bars), all treatment groups were stored only in 0% DE. Across all panels within (A) or within (B), means with the same letter do not differ significantly (Tukey multiple comparison adjustment).

Adult beetles of different initial ages produced eggs during all census intervals at all DE concentrations (Fig. 3). Net egg production from each 5-day census interval was standardized/female/day, log-transformed, and analyzed using PROC MIXED with an autoregressive covariance structure, DE concentration and beetle age as fixed effects, and census interval as the repeated measure. The 3-way interaction among DE concentration, beetle age and census time was not significant (F_28,189_ = 1.16, p = 0.278), nor was the 2-way interaction between DE concentration and beetle age (F_4,27_ = 1.64, p = 0.194). There was a significant 2-way interaction between beetle age and census time (F_14,189_ = 12.20, p<0.001). Pairwise comparisons indicated that this interaction resulted from significantly-lower *Nt* for newly-emerged beetles (0 weeks old) during the first census, whereas *Nt* was indistinguishable among beetle age groups from the day 10 census onwards (Fig. 3A). There was also a significant 2-way interaction between DE concentration and census time (F_14,189_ = 3.79, p<0.001). Pairwise comparisons (Fig. 3B) indicated that there was significantly higher *Nt* in one or both of the DE treatments compared to the 0% DE control at each census up to day 25. However, following the return of all beetles to DE-free medium, the *Nt* of beetles formerly in DE declined and for the remaining censuses became indistinguishable from the 0% DE control for the corresponding census period.

### Effect of DE on Egg Cannibalism by Adults

#### Experiment 2

Mortality was negligible. Only 2/210 females and 6/210 males died. Data from affected vials were adjusted to express results/female/day. Note that in the following descriptions of results, although all groups of beetles were stored in 0% DE during the baseline round, they are referred to by the DE concentration that they would receive in the treatment round.

**Figure 4:**
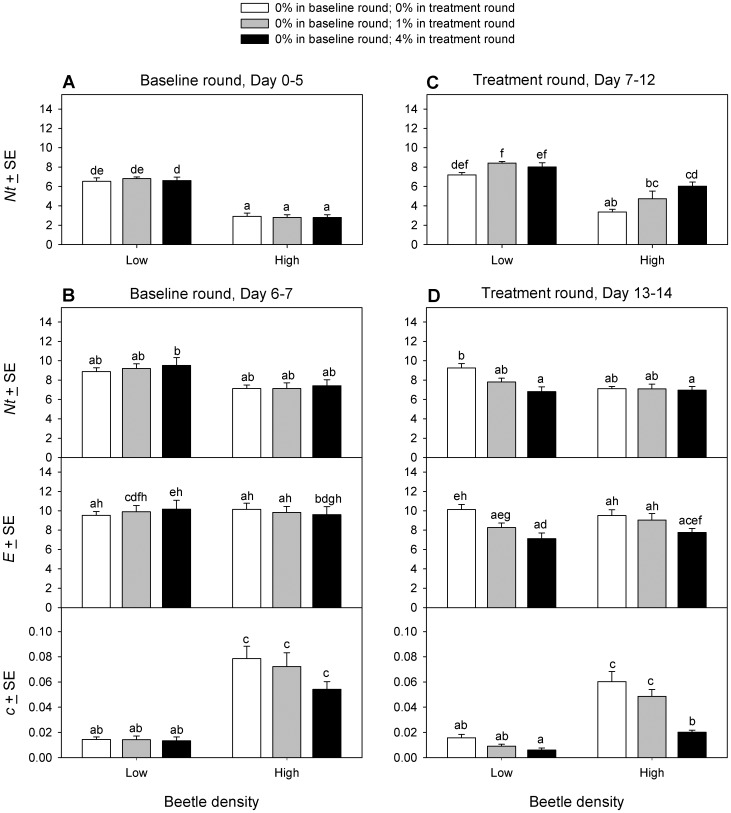
Net fecundity, real fecundity and egg cannibalism of *Tribolium confusum* exposed to diatomaceous earth. Fecundity of groups of adult beetles at low density (10 beetles/12 g medium) or high density (10 beetles/3 g medium) determined in a series of censuses done sequentially over a 14-day period (experiment 2). During the baseline round, all beetles were in 0% DE. First, net fecundity (*Nt*) was estimated in a 5-day-long census (A). Following a 1-day-long transition period, a 1-day-long cannibalism assay was done (B) that determined *Nt*, real fecundity (*E*) and cannibalism rate (*c*) in the absence of DE. During the treatment round, the beetles from the baseline round were stored at the same densities but in different concentrations of diatomaceous earth (DE). Then, *Nt* was estimated in a second 5-day-long census (C), followed by a second 1-day-long cannibalism assay (D) that determined *Nt*, *E* and *c* in the presence of DE. Within each pair of baseline and DE treatment observations, means with the same letter do not differ significantly (Tukey multiple comparison adjustment).

Net egg production from the 5-day census that started the baseline and treatment rounds (Fig. 4A, C) was analyzed using PROC MIXED with a compound symmetry covariance structure, DE concentration and beetle density as fixed effects, and observation round (baseline vs. treatment) as the repeated measure. The 3-way interaction among DE concentration, beetle density and observation round was significant (F_2,36_ = 8.09, p = 0.001). Pairwise comparisons revealed several patterns. During the baseline round, *Nt* was uniformly high in all low density groups, and 40% lower in all high density groups (Fig. 4A). During the treatment round, beetles at low density had *Nt* similar to baseline in 0% DE, but *Nt* was elevated significantly during DE exposure, about 1.2× baseline for both 1% and 4% DE (Fig. 4C). During the treatment round, beetles at high density also had *Nt* similar to baseline in 0% DE, and *Nt* was also elevated significantly during DE exposure, but this time by about 1.7× baseline in 1% DE and 2.1× baseline in 4% DE (Fig. 4C). By way of comparison, the average increase in *Nt* in 4% DE over 0% DE from other 5-day censuses at high density was 1.6× in both experiment 1a (Fig. 2) and experiment 1b (Fig. 3B).

In the cannibalism assay proper (Fig. 4B, D), each variable was analyzed using PROC MIXED, with unstructured covariance, DE concentration and beetle density as fixed effects, and observation round (baseline vs. treatment) as the repeated measure.

Net fecundity showed a significant 3-way interaction among DE concentration, beetle density and census time (F_2,36_ = 5.58, p = 0.008). Pairwise comparisons (Fig. 4B) revealed no difference among any combinations of beetle density and DE concentration in the baseline round. The overall *Nt* at low density declined after DE was applied, but this was only significant and most evident at the highest (4% DE) concentration (Fig. 4D). By contrast, at high density, *Nt* in the treatment round remained similar to *Nt* in the baseline round and was independent of DE concentration.

Real fecundity (*E*) showed a significant 3-way interaction among DE concentration, beetle density and census time (F_2,36_ = 4.02, p = 0.027). Pairwise comparisons showed no difference in *E* among beetle densities or DE concentrations during the baseline round (Fig. 4B). Real fecundity at low density remained similar to baseline values for beetles kept in 0% DE during the treatment round, whereas *E* declined significantly relative to baseline in a concentration-dependent manner on exposure to 1% or 4% DE, to as little as 0.7× baseline (Fig. 4D). Real fecundity at high density declined significantly relative to baseline only on exposure to 4% DE, where *E* was 0.8× baseline (Fig. 4D).

Cannibalism rate (*c*), expressed as the proportion of eggs eaten/beetle/day, was transformed for analysis using the angular transformation. The 3-way interaction among DE concentration, beetle density and census time was not significant (F_2,36_ = 0.51, p = 0.608). However, the interaction between beetle density and observation round was significant (F_2,36_ = 10.9, p = 0.002) as was the interaction between DE concentration and observation round (F_1,36_ = 5.08, p = 0.011), and the interaction between beetle density and DE concentration was marginal (F_2,36_ = 2.94, p = 0.066). Therefore, pairwise comparisons were done for all combinations of the 3 factors (Fig. 4B, D). In the baseline round, *c* did not differ among DE concentrations at low or high beetle densities, but overall *c* was 4.5× greater at high density than at low density (Fig. 4B). In the treatment round (Fig. 4D), at low density, *c* was indistinguishable from baseline values and unaffected by the addition of DE. At high density, the highest concentration (4%) of DE reduced *c* significantly (Fig. 4D), to 0.4× baseline.

### Population Growth in the Presence of DE

#### Experiment 3

**Figure 5:**
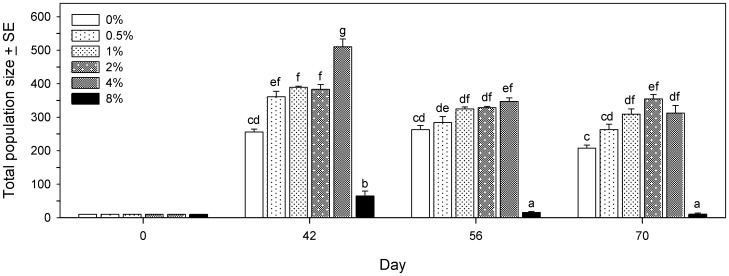
Population growth of *Tribolium confusum* exposed to diatomaceous earth. Total numbers of live beetles (larvae, pupae and adults) present at different times after introducing 10 beetles into culture tubes containing different concentrations of diatomaceous earth (DE) (experiment 3). Means with the same letter do not differ significantly (Tukey multiple comparison adjustment).

The population size of live beetles (larvae, pupae and adults combined) at 3 time periods and 6 DE concentrations was analyzed as a factorial design using PROC GLM, with time and DE concentration as fixed effects. The interaction between time and DE concentration was significant (F_10,54_ = 4.77, p<0.001). Pairwise comparisons (Fig. 5) showed that control beetles (0% DE) had similar total population sizes at days 42, 56 and 70. At day 42, as little as 0.5% DE resulted in a significant increase in population size, which continued to increase to a maximum in 4% DE, but then dropped markedly for beetles that were in 8% DE. The same pattern was observed at days 56 and 70, but peak population sizes did not reach the levels seen at day 42, and populations in 8% DE were significantly smaller than at day 42.

**Figure 6:**
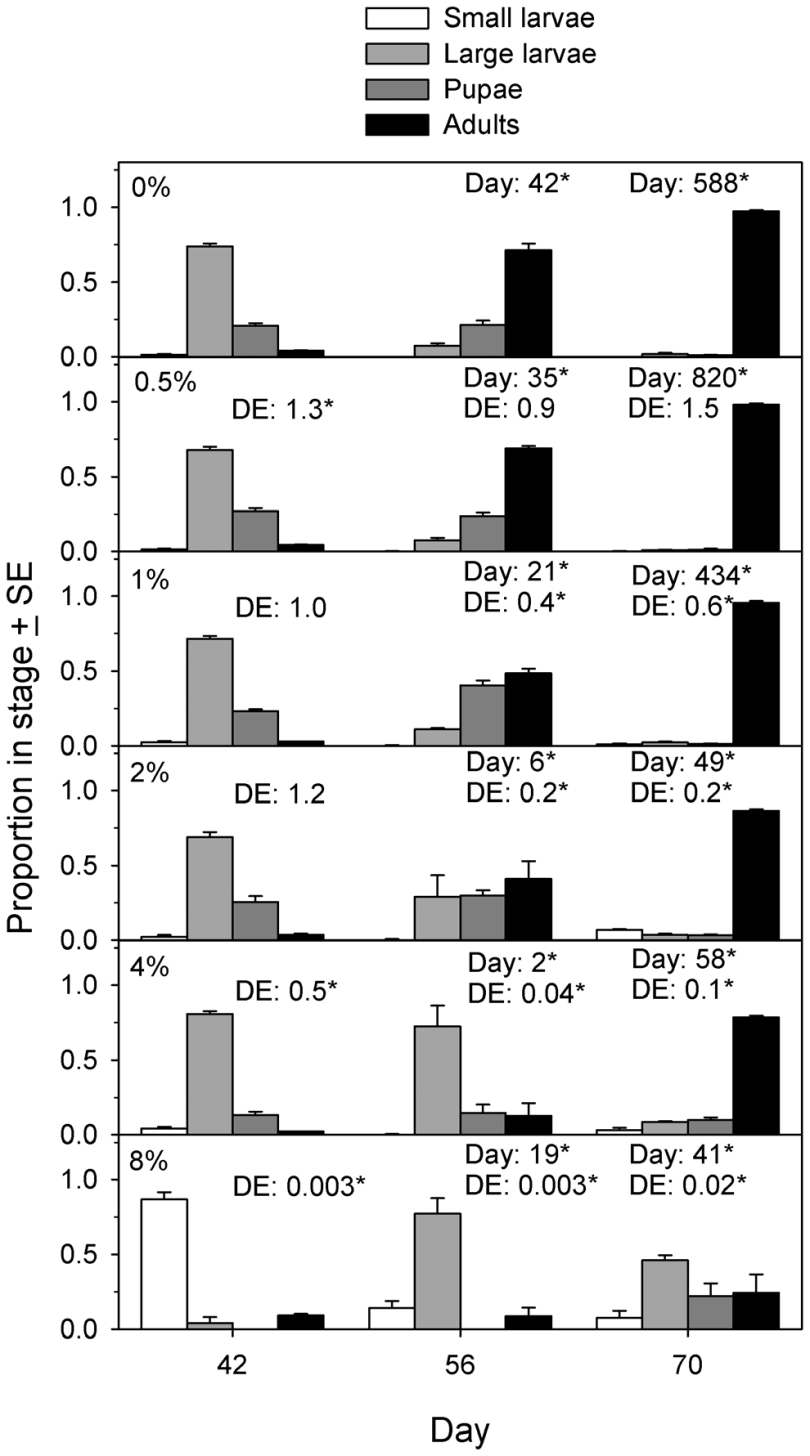
Population structure of *Tribolium confusum* exposed to diatomaceous earth. Proportion of live beetles in different developmental stages present at different times after introducing 10 beetles into culture tubes containing different concentrations of diatomaceous earth (DE) (experiment 3). Numbers above each group of bars are odds ratios calculated from ordered logistic regressions comparing the distribution of stages in that group to a reference group (for “Day”, the reference group was the day 42 observation for the same DE concentration; for “DE”, the reference group was the 0% DE treatment for the same day). *Odds ratio significantly different from 1.

Beetle developmental stage structure varied with time and DE concentration (ordered logistic regression, day×DE interaction effect, Wald χ^2^ = 771, p<0.001). On day 56 and day 70, beetles were more likely to be in a later stage than at day 42 (odds ratio >1) at each DE concentration (Fig. 6). On the other hand, as DE concentration increased beetles were more likely to be in an earlier developmental stage compared to 0% DE (odds ratio <1); at day 42 the odds ratio was ∼1 at ≤2% DE but declined for ≥4% DE, whereas on day 56 and day 70 significant declines in odds ratios occurred for ≥1% DE (Fig. 6). Populations in 0% and 0.5% DE followed a similar time course. They were dominated by large larvae (>65%) on day 42 and by adults (>65%) on day 56, and comprised adults almost exclusively (>97%) by day 70. At 1–4% DE, the most striking difference from this pattern occurred on day 56, where high proportions (up to 70%) were still large larvae (Fig. 6). However, by day 70 adults (>75%) again predominated. Population composition in 8% DE differed markedly (Fig. 6). On day 42, 87% of beetles were small larvae, and by day 70 large larvae (45%) still predominated, with the first pupae and new adults observed.

### Effect of DE on Egg Hatching Success

#### Experiment 4

Eggs laid in medium containing DE (experiment 4a) had similar hatching success in 0–8% DE (Fisher’s exact test, p = 0.33), 93.7% of 143 eggs overall. Although hatching success was consistent in ≤8% DE, survival of hatched larvae on the day 10 observation differed. Whereas 99% of 100 larvae in 0–4% DE were alive on day 10, only 50% of 32 larvae in 8% DE were alive (Fisher’s exact test, p<0.001). Eggs laid in DE-free medium and then transferred into medium containing DE (experiment 4b) also showed no difference among DE treatments in the proportion hatching in the first trial (Fisher’s exact test, p = 1.00), with 91.7% of 72 eggs hatching overall, or among DE treatments in the second trial (Fisher’s exact test, p = 0.671), with 91.0% of 144 eggs hatching overall. Hatching success in these 2 experiments was compared using logistic regression in a factorial design (factor 1: DE at 0%, 1% or 4%; factor 2: experiment 4a or 4b) with interactions. There was no effect of DE (Wald χ^2^ = 0.89, p = 0.64) or experiment (χ^2^ = 0.66, p = 0.72), and there was no interaction (χ^2^ = 1.42, p = 0.84). Thus, pooling data across experiments and 0–4% DE, only 8.0% (95% CI = 5.3–11.6%) of eggs failed to hatch.

### Effect of DE on Egg Cannibalism by Larvae

#### Experiment 5

Cannibalism rate *c* (following angular transformation) was analyzed in a factorial design using PROC GLM, with larval size category and DE concentration as fixed effects. There was no interaction between larval size and DE concentration (F_1,30_ = 0.31, p = 0.735), and the larval size main effect was not significant (F_2,30_ = 2.35, p = 0.112). However, the main effect of DE concentration was significant (F_1,30_ = 6.20, p = 0.019); the rate of cannibalism was significantly lower in larvae exposed to 4% DE (mean *c* = 0.009, SE = 0.002, n = 18) compared to larvae in 0% DE (mean *c* = 0.028, SE = 0.006, n = 18).

### Survival of a Cohort of Eggs Under Various Egg Cannibalism Scenarios

#### Experiment 6a

Of the 24 initial eggs in each DE treatment, there were 2 eggs in 0% DE that did not hatch, and 1 larva lost during handling, resulting in 21 that reached adult. There were 2 eggs in 1% DE that did not hatch, 1 larva found dead 2 days post-hatching, and 3 larvae lost during handling, resulting in 18 that reached adult. There were 2 eggs in 4% DE that did not hatch, 1 larva found dead 23 days post-hatching, and 5 larvae lost during handling, resulting in 16 that reached adult. Over all DE treatments, excluding handling losses, 96% of 57 larvae that hatched developed into adults.

**Figure 7:**
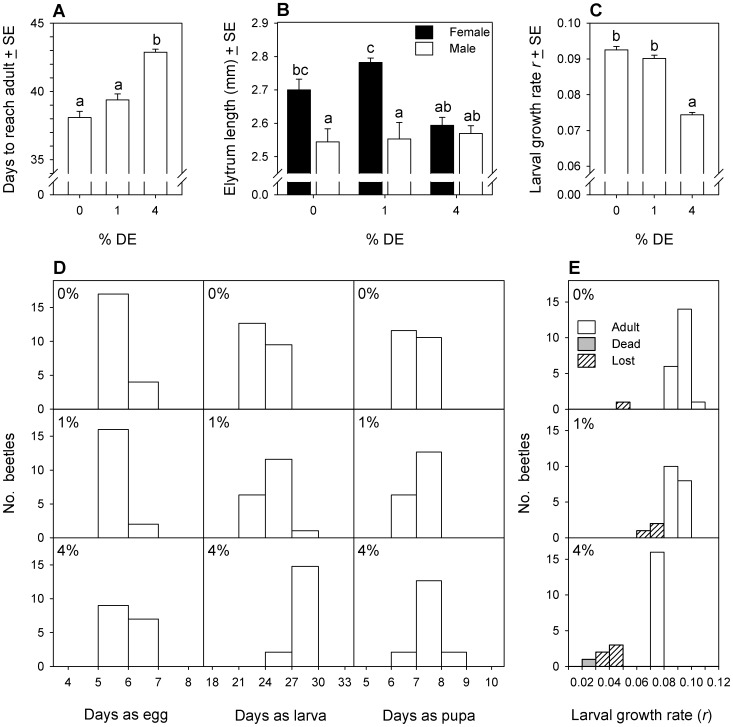
Development and growth of a cohort of *Tribolium confusum* eggs exposed to diatomaceous earth. Result of daily observations on a cohort of eggs stored continuously in different concentrations of diatomaceous earth (DE) (experiment 6a). (A) Days required from egg deposition to adult emergence. (B) Body size of adult male and female beetles, as determined by length of the left elytrum. (C) Growth rate coefficients for larval length calculated from beetles that completed development to adult. (D) Days spent as egg, larva and pupa for beetles that reached adult. (E) Comparison of growth rate coefficients for larval length among beetles that completed development to adult, died during larval development, or were lost during larval handling. Within each panel, means with the same letter do not differ significantly (Tukey multiple comparison adjustment).

The length of time between egg deposition and molt to adult differed according to DE concentration (ANOVA, F_2,49_ = 38.9, p<0.001), but not beetle sex (F_1,49_ = 2.2, p = 0.147) and there was no interaction (F_2,49_ = 2.65, p = 0.081). Development took significantly longer in 4% DE than in 0% or 1% DE, by about 4 days (Fig. 7A). Measurements of elytrum length revealed a significant interaction between beetle sex and DE concentration (ANOVA, F_2,49_ = 4.7, p = 0.013). Elytrum length of males did not vary with DE concentration, whereas females that developed in 0% or 1% DE had longer elytra than females that developed in 4% DE (Fig. 7B). Larvae grew in length exponentially until they molted to the 7^th^ larval stage. Lengths of each individual over time were used to determine its growth rate, *r*, according to the equation: *x_t_ = x_0_* (1*+r*)*^t^*, where *x* is the length in mm and *t* is the age of the larva in days. Growth rates of larvae differed among DE treatments (ANOVA, F_2,52_ = 119, p<0.001) but not sex (F_1,49_ = 0.05, p = 0.830) and there was no interaction (F_2,49_ = 0.52, p = 0.600). Pairwise comparisons on larvae that completed development revealed that larvae had lower growth rates in 4% DE than larvae in 0% DE or 1% DE (Fig. 7C). Increasing DE concentration delayed egg hatching (Mantel-Haenszel test, χ^2^_1_ = 4.04, p = 0.046), larval development (χ^2^_1_ = 33.3, p<0.001) and pupal development (χ^2^_1_ = 8.46, p = 0.003) (Fig. 7D).

The larvae that were lost during handling were always those with the lowest growth rates, particularly in 4% DE (Fig. 7E). At the time of loss, larvae averaged 2.0±0.25 (SE) mm long. This was interpreted as evidence of an increased risk of larval loss through daily handling of the smallest larvae, and was a basis for the decision to census the cohorts less frequently in experiments 6b–d, and to delay the initial census until day 14, by which time the average larva would already be 2 mm long.

#### Experiment 6b

**Figure 8:**
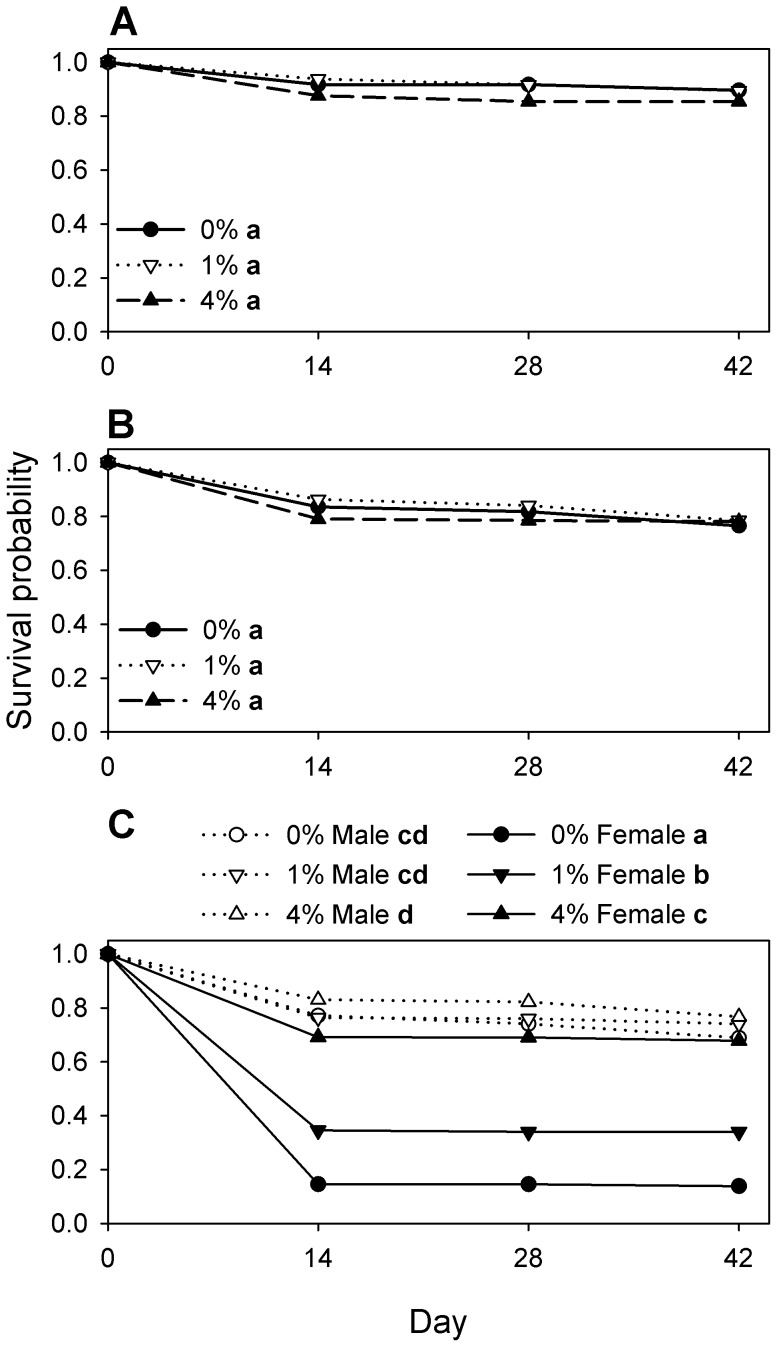
Survival of a cohort of *Tribolium confusum* eggs exposed to diatomaceous earth. Life table survival functions for a cohort of eggs stored continuously in different concentrations of diatomaceous earth (DE) and under different scenarios for potential cannibalism. (A) Eggs stored individually, to prevent cannibalism (experiment 6b). (B) Eggs stored in groups of 100, permitting cannibalism only by offspring from those eggs (experiment 6c). (C) Eggs stored in groups of 200 plus 10 introduced virgin adult males or virgin adult females, permitting cannibalism by either the offspring from those eggs or by the introduced adults (experiment 6d). Within each panel, means with the same letter do not differ significantly (Tukey multiple comparison adjustment).

**Figure 9:**
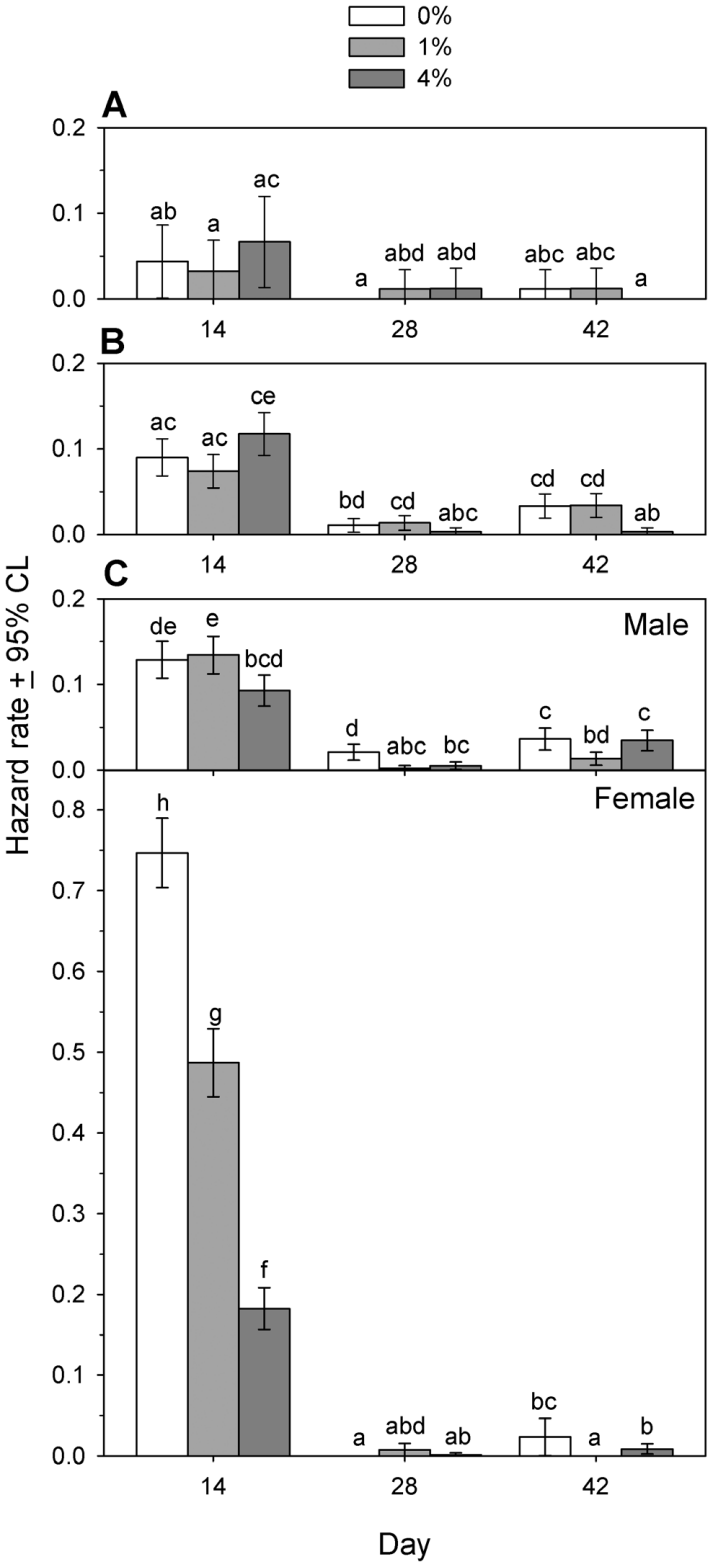
Hazard rates for a cohort of *Tribolium confusum* eggs exposed to diatomaceous earth. Hazard rates over 14-day intervals as determined by life table analysis of cohorts of eggs stored continuously in different concentrations of diatomaceous earth (DE) and under different scenarios for potential cannibalism. (A) Eggs stored individually, to prevent cannibalism (experiment 6b). (B) Eggs stored in groups of 100, permitting cannibalism only by offspring from those eggs (experiment 6c). (C) Eggs stored in groups of 200 plus 10 introduced virgin adult males or virgin adult females, permitting cannibalism by either the offspring from those eggs or by the introduced adults (experiment 6d). Within each day, columns with the same letter from any DE concentration or experiment have overlapping 95% CI.

Survivorship of a cohort of eggs maintained individually was independent of DE concentration (log rank test of equality over strata, χ^2^_2_ = 0.54, p = 0.76) (Fig. 8A). The hazard rate was low throughout the experiment (Fig. 9A). At the first census on day 14, mortality in all DE levels was due almost exclusively to failure to hatch (Table 1). Cannibalism was not possible, and handling losses in this experiment were 0% of 131 (95% CI = 0–2.8%) hatched larvae. The absence of handling losses and narrow confidence interval in this experiment, in which the first census was delayed until day 14, was the basis for assuming that handling losses were negligible (Table 1) in subsequent experiments (6c–d) that also had their first census on day 14.

**Table 1:** Composition of *Tribolium confusum* cohorts 14 days after egg deposition.

Experiment	% DE	*L*	*U*	*D*	*M*	*C*
6b	0	0.917	0.062	0.021	0	0
	1	0.917	0.083	0	0	0
	4	0.875	0.125	0	0	0
6c	0	0.835±0.015	0.080	0±0	0	0.085±0.015^ab^
	1	0.862±0.018	0.080	0.005±0.006	0	0.052±0.015^a^
	4	0.790±0.007	0.080	0.008±0.005	0	0.122±0.010^ab^
6d(males)	0	0.772±0.051	0.080	0±0	0	0.148±0.051^ab^
	1	0.763±0.045	0.080	0±0	0	0.157±0.045^ab^
	4	0.830±0.043	0.080	0±0	0	0.090±0.043^ab^
6d(females)	0	0.145±0.055	0.080	0±0	0	0.775±0.055^d^
	1	0.345±0.027	0.080	0.007±0.002	0	0.568±0.026^c^
	4	0.692±0.022	0.080	0±0	0	0.228±0.022^b^

Eggs and the larvae that hatched from them were maintained in different concentrations of diatomaceous earth (DE) and in experimental designs producing different potential for cannibalism. Data represent proportions of the initial egg cohort recorded on a day 14 census as live larvae (*L*), unhatched eggs (*U*), dead larvae (*D*), missing from handling loss (*M*), and cannibalized (*C*). Initial cohort composition– experiment 6b: individual eggs, n = 48/DE treatment; experiment 6c: n = 4 replicates of 100 eggs/DE treatment; experiment 6d: n = 3 replicates of 200 eggs, plus 10 virgin adult males or females,/DE treatment. Values are mean ± SE of replicate determinations. Cannibalism estimate means with the same letter do not differ significantly (Tukey multiple comparison adjustment).

#### Experiment 6c

Survivorship of a cohort of eggs maintained in groups of 100 was independent of DE concentration (log rank test of equality over strata, χ^2^_2_ = 0.49, p = 0.78) (Fig. 8B). While the hazard rate was generally low throughout the experiment, non-overlapping confidence intervals indicated that it was higher during the first 14 day interval (Fig. 9B). The presence of larvae in this experiment posed a risk of egg cannibalism that prevented complete recovery and characterization of eggs for hatching success, so the pooled hatching success from experiment 4 was used as the basis for assuming a constant 8% failure to hatch for eggs in all DE concentrations (Table 1). Few or no dead beetles were found, and under the assumption of no handling loss, the remaining beetles unaccounted for were assumed to be lost to cannibalism, with proportion cannibalized *C* = 1– *L* – *U* – *D* – *M* (Table 1). In this experiment, the proportion lost from the cohort due to cannibalism was similar to losses from hatching failure (Table 1).

#### Experiment 6d

Survivorship of a cohort of eggs maintained in groups of 200, in the continuous presence of virgin adults, varied according to DE concentration and sex of the adult beetles present (log rank test of equality over strata, χ^2^_5_ = 908, p<0.001). Survival in the presence of virgin males was high and did not vary with DE concentration, whereas survival in the presence of females was lowest in 0% DE, but increased significantly in 1% DE and increased again in 4% DE (Fig. 8C). Survival in the presence of females was lower than in the presence of males at all DE concentrations tested (Fig. 8C). Again, the bulk of mortality in the presence of adult males or females occurred during the first 14 days (Fig. 9C). No dead beetles were found, and, retaining the assumption of no handling loss and an expected 8% failure to hatch, DE concentration and sex of adult beetle both affected cannibalism (Table 1). For males, cannibalism accounted for 65% of the cohort losses in 0% DE and 70% of losses in 1% DE, whereas cannibalism in 4% DE was on par (53% of losses) with failure to hatch, as was seen in experiment 6c (Table 1). For females, cannibalism accounted for 91% of the losses in 0% DE, 88% of the losses in 1% DE, and 74% of the losses in 4% DE (Table 1).

Although hazard rates exhibited sporadic differences among experiments on days 28 and 42 (Fig. 9), the day 14 observations were most relevant to the fate of an initial cohort of eggs. Rearing of eggs individually or in groups resulted in similar day 14 hazard rates at all DE concentrations (Fig. 9A, B). The addition of adult males (Fig. 9C) resulted in higher hazard rates in the absence of DE, but 4% DE reduced hazard rates (Fig. 9C) to levels seen in the absence of adult beetles (Fig. 9A, B). The addition of adult female beetles (Fig. 9C) was associated with a markedly higher hazard rate in 0% DE than when in the presence of adult males (Fig. 9C) or in the absence of adult beetles (Fig. 9A, B). Although hazard rates in the presence of adult females declined as DE concentration increased, in 4% DE they still remained higher than those observed in the presence of males (Fig. 9C) or in the absence of adults (Fig. 9A, B).

The use of replicate trials in experiments 6c and 6d permitted an independent estimate of losses due to cannibalism for each replicate. These estimates (Table 1) were analyzed using PROC GLM in a factorial design. There was a significant interaction between experiment and DE treatment (F_4,21_ = 29.1, p<0.001). The differences in hazard rates among DE concentrations and experiments (Fig. 9) were paralleled by differences in cannibalism estimates (Table 1); the presence of adult females resulted in the greatest cannibalism losses in 0% DE, with cannibalism reduced as DE concentration increased.

Not only were few dead beetles found in any of the day 14 censuses, it was uncommon to find a dead beetle of any developmental stage even by the end of the experiments on day 42. Collectively, in experiments 6b–d, there were 4944 eggs used initially, and 3051 live beetles present at the end, leaving 1893 dead or absent. The carcasses of only 18 larvae (1.0% of the dead or absent beetles; mean length = 1.56 mm, SE = 0.22), 1 pupa (0.05%), and 73 adults (3.8%) were found. A high proportion (92.3%) of the 3192 larvae that were present on day 14 in all DE treatments in experiments 6b–d, completed their development into adults, similar to experiment 6a (Fisher exact test, p = 0.32).

## Discussion

The first aim of this study was achieved by observing an increase in net fecundity of *T. confusum* following exposure to sublethal (≤4%) concentrations of DE over a broad range of test conditions; this effect was abrogated upon removal from DE and was independent of beetle age. The second aim of this study was achieved by failing to support the hypothesis that DE increased net fecundity by increasing oviposition – oviposition was actually reduced – and by supporting the hypothesis that DE acted by reducing egg cannibalism. The third aim of this study was achieved by demonstrating that, in undisturbed cultures, continuous exposure to sublethal DE concentrations resulted in a sustained increase in population size, and that this increase can be explained largely by reduced egg cannibalism by adult females. These findings represent the first report that DE inhibits egg cannibalism by adult and larval insects.

At high concentrations, DE was lethal to both adults and larvae, as expected from other studies [38]. In addition to addressing its central aims, the present study is the first report that DE also has a complex suite of sublethal effects on this insect. Some of these sublethal effects could be predicted to have negative consequences for the beetle: in addition to reduced oviposition, DE caused a delay in development to adult stage, lower growth rate of larvae, and the production of smaller adult females. Female mass is unrelated to net fecundity in many species of *Tribolium*, but is positively correlated in at least one strain of *T. confusum* [39]. Other properties that were measured were more neutral to the presence of sublethal DE: in addition to minimal effects on adult mortality, egg hatchability (even when coated directly with DE-containing medium) and larval survival to adult stage were unaffected. Finally, sublethal DE promoted population growth by reducing cannibalism on eggs by larvae and adults, particularly female adults.

In the absence of the direct observation of ingestion of one individual by another, cannibalism, by its nature, must be inferred by the disappearance of individuals known to be present previously and whose disappearance cannot be explained by other means. The cannibalism assays achieved this by documenting loss of marked eggs in the presence of adults, or loss of unmarked eggs in the presence of larvae, while demonstrating that all eggs remained in the absence of other beetles. The cohort studies raised the possibility that some disappearance of small, slow-growing larvae might result from handling loss. However, delaying census times resolved this concern. The high proportion of larvae that reached adult even in the presence of other larvae or adults supports previous observations [7] and indicates that cannibalism on larvae was not a major mortality factor in the experiments that were done. Therefore, the cohort studies support the conclusion that reduced egg cannibalism by adult females was the major contributor to increased population growth. The suppression of egg cannibalism by sublethal DE more than counteracted its other negative effects on *T. confusum*. Both male and female adult *T. confusum* cannibalize eggs; females at a greater rate than males [6], [11]. One study suggests that the difference between sexes for *T. confusum* is more pronounced after mating, and that egg cannibalism in both sexes declines after mating, which in conjunction with kin discrimination may reduce filial cannibalism [12]. This conclusion may not apply generally. Strain and species differences in cannibalism behavior are known within the genus *Tribolium* [6], [7]. Indeed, the present study revealed similar patterns of cannibalism, and similar effects of DE, whether using mated or virgin beetles.

Egg cannibalism in *Tribolium* spp. has been known for over 80 years [4]. Many studies have demonstrated that the effectiveness of egg cannibalism as a population regulation mechanism can be modified by various extrinsic and intrinsic factors. However, this is the first study to explicitly investigate the effects of DE on egg cannibalism and its consequences for population growth. In general, cannibalism may be influenced by starvation, population density, stress, and availability of victims [2] and these modifying factors may interact in a complex fashion [6], [7], [11]. Cannibalism rates are high and relatively constant with age in *T. castaneum* up to at least 99 days old [6] and this supports the interpretation that suppression of cannibalism, which was confirmed in 2–4 week old *T. confusum* in the present study, contributed to the general increase in net fecundity that was observed in all age groups. The known action of DE on insects is through desiccation [17] and DE is more effective at low relative humidity [22], [40], so the low ambient relative humidity in the present study provided an environment where DE might be expected to have a potent desiccating effect. However, egg cannibalism by the beetle *Chilocorus nigritus* appears unaffected by a range of humidity [41], and studies on *T. confusum* suggest that cannibalism on eggs is used to obtain nutrients [9], [14] and not water [10]. Indeed, rates of cannibalism in the presence of the drying effects of DE actually decreased. However, nutritional stress alone is not sufficient to stimulate cannibalism. For example, parasitized beetles, in which nutritional stress is presumed to be occurring, do not have a uniform cannibalism response. While parasitism by larvae of the tapeworm *Hymenolepis diminuta* does not affect egg cannibalism by *T. confusum*, the cannibalism rate increases in parasitized *T. castaneum* [15]. The effect of DE on egg cannibalism is clearly not a generalized stress response, but seems to depend on the specific nature of the stress. Despite a long history of egg cannibalism research on this insect, and the many modifying factors that have been identified, the underlying physiological mechanism of this behavior has not been determined.

Most published studies on DE have been done from a pest management perspective, and as such have focused on the determination of lethal doses [17], [20], [21]. Moreover, studies of reproductive effects of DE are much less common than studies of its effects on insect mortality. This study explicitly used low concentrations of DE to investigate sublethal effects specifically with regard to beetle reproduction and cannibalism behavior. Other reproductive studies used only lethal DE concentrations and monitored progeny after the removal of adults [21]–[23], [42], thereby removing a potential source of egg cannibalism and focusing only on oviposition. These studies show that beetles surviving low levels of DE exposure can still produce offspring, but only at levels comparable to, or lower than, non-exposed beetles. Some of these studies used beetles that are not known egg cannibals, and the one study done on *T. confusum* [42] reports only qualitatively that offspring were still produced during DE exposure. There are occasional reports that sublethal pesticide exposure can increase arthropod fecundity by increasing oviposition [30], and that environmental stress in the form of parasitism can cause an increase in the fecundity of some arthropods, molluscs and fish [43]–[45]. In the present study, however, it appears that sublethal DE acted as a more typical environmental stressor by reducing oviposition of *T. confusum*. The results suggest that a broader examination of the role of cannibalism suppression in pest management scenarios is warranted.

Consideration of the known modifying effects on egg cannibalism may provide an explanation for one of the apparently counterintuitive results of the present study: the lack of increased net fecundity following DE exposure during a 1-day cannibalism assay, compared to the consistent increase that was observed when 5-day-long census periods were used (experiment 1). It has been hypothesized [6] that the eating of an egg encountered by a beetle is a specific behavioral response that increases in probability as the frequency of egg encounters increases. Over a short period, such as 1 day, there would be few newly-laid eggs, and even with the dyed eggs added during the cannibalism assays the total egg density may have been too low to stimulate strong cannibalism. Net fecundity measured over just 1 day would be dominated by the reduced oviposition rate. Over a longer period, such as 5 days, newly-laid eggs would steadily accumulate and the increasing total egg density would provide increasing stimulus for cannibalism. Measured over longer periods, net fecundity would become dominated more by cannibalism effects. Thus the inhibitory effects of DE on egg cannibalism detected even during the 1-day cannibalism assay could be speculated to be indicative of an even greater ability to increase net fecundity in longer census intervals, or in other undisturbed situations.

A broader implication of these findings, and one initial concern that prompted the present study, is that improper application of DE as an insecticide might actually increase pest populations [24]. A potential long-term consequence of applying DE-based insecticides in an open system, whether in an agricultural or domestic setting, is that zones of sublethal DE may be generated that could inhibit egg cannibalism. These zones might occur in areas peripheral to application sites, or as a result of disturbance or wind action. On the one hand, transitory exposure of *T. confusum* to DE in these peripheral zones can now be predicted to inhibit their cannibalism behavior. On the other hand, cannibalism rates for *T. confusum* specifically were relatively low to begin with at low beetle density, corroborating previous work [6] and leaving little room for further depression by sublethal DE, so the initial concern does not seem to be supported for this particular pest species. However, the importance of cannibalism in open systems for *Tribolium* spp., or for insects in general, is not well known; almost all of the literature on cannibalism in non-carnivorous insects comes from laboratory studies [3].

A number of questions were raised that will require broader study. The generality of the ability of DE to inhibit egg cannibalism has yet to be determined. The lethal concentration varies among DE sources [17], [46], among species [40], and even among strains of the same species of insect [46]; these sources of variation are likely to influence the sublethal effects of DE on egg cannibalism by both adults and larvae. Furthermore, depending on its source, DE can differ in pH, SiO_2_ content, particle shape and size, and types of non-SiO_2_ solids [17]; if these have nutritional or other physiological effects on the beetle that influence cannibalism, then its potential to inhibit cannibalism may depend on certain DE-source×beetle strain combinations. One approach for studying cannibalism has been through the identification and breeding of high and low cannibalism strains of insects [9], [10]. Sublethal DE provides an ability to manipulate cannibalism behavior directly without altering the genetic background of the study animal or external conditions such as animal density. The precise physiological and genetic pathways that control egg cannibalism are not well understood. However, the strong and rapid inhibition of egg cannibalism by DE shown in the present study, at levels that do not themselves substantially affect mortality, makes DE an ideal investigative tool for such studies. Finally, from an evolutionary perspective, the appearance of cannibalism is often viewed as a behavior that has evolved to prevent population increases where high densities might exacerbate the effects of stressful environmental conditions experienced by an organism [1], [2], [14], [47]. We see in *T. confusum* the opposite response, in which a mild environmental stress suppresses the organism’s normal population regulation mechanism and allows its population to increase. This type of response may be adaptive in situations where the environmental stress may precede a density-independent population reduction, such as in the case of exposure to a pesticide or other environmental toxin.
